# The Hippo effector TAZ (WWTR1) transforms myoblasts and TAZ abundance is associated with reduced survival in embryonal rhabdomyosarcoma

**DOI:** 10.1002/path.4745

**Published:** 2016-08-22

**Authors:** Abdalla Mohamed, Congshan Sun, Vanessa De Mello, Joanna Selfe, Edoardo Missiaglia, Janet Shipley, Graeme I Murray, Pete S Zammit, Henning Wackerhage

**Affiliations:** ^1^ School of MedicineDentistry and NutritionUniversity of AberdeenUK; ^2^ Randall Division of Cell and Molecular BiophysicsKing's College LondonUK; ^3^ Sarcoma Molecular Pathology Team, Divisions of Molecular Pathology and Cancer TherapeuticsInstitute of Cancer ResearchLondonUK; ^4^ Institut Universitaire de Pathologie de Lausanne IUPSwitzerland

**Keywords:** Hippo pathway, TAZ, WWTR1, embryonal rhabdomyosarcoma, myoblasts

## Abstract

The Hippo effector YAP has recently been identified as a potent driver of embryonal rhabdomyosarcoma (ERMS). Most reports suggest that the YAP paralogue TAZ (gene symbol WWTR1) functions as YAP but, in skeletal muscle, TAZ has been reported to promote myogenic differentiation, whereas YAP inhibits it. Here, we investigated whether TAZ is also a rhabdomyosarcoma oncogene or whether TAZ acts as a YAP antagonist. Immunostaining of rhabdomyosarcoma tissue microarrays revealed that TAZ is significantly associated with poor survival in ERMS. In 12% of fusion gene‐negative rhabdomyosarcomas, the TAZ locus is gained, which is correlated with increased expression. Constitutively active TAZ S89A significantly increased proliferation of C2C12 myoblasts and, importantly, colony formation on soft agar, suggesting transformation. However, TAZ then switches to enhance myogenic differentiation in C2C12 myoblasts, unlike YAP. Conversely, lentiviral shRNA‐mediated TAZ knockdown in human ERMS cells reduced proliferation and anchorage‐independent growth. While TAZ S89A or YAP1 S127A similarly activated the 8XGTIIC–Luc Hippo reporter, only YAP1 S127A activated the Brachyury (T‐box) reporter. Consistent with its oncogene function, TAZ S89A induced expression of the ERMS cancer stem cell gene Myf5 and the serine biosynthesis pathway (Phgdh, Psat1, Psph) in C2C12 myoblasts. Thus, TAZ is associated with poor survival in ERMS and could act as an oncogene in rhabdomyosarcoma. © 2016 The Authors. *The Journal of Pathology* published by John Wiley & Sons Ltd on behalf of Pathological Society of Great Britain and Ireland.

## Introduction

Yap (gene symbol *Yap1*) and Taz (gene symbol *Wwtr1*) are transcriptional co‐factors that regulate gene expression mainly by binding Tead1–4 1, 2. Yap and Taz are not only regulated by the Hippo pathway 3 but are also additionally targeted by a network of other signalling molecules, including *PIK3CA* 4, *KRAS* 5, 6, 7 and *CTNNB1* (encoding β‐catenin) 8. Importantly, these crosstalking genes are affected by recurrent pan‐cancer mutations 9, including in embryonal rhabdomyosarcoma (ERMS) 10.

Persistent Yap hyperactivity, as a consequence of expressing a constitutively active *YAP1 S127A* mutant, results in tumours of the liver 11 and skin in mice 12. Constitutive Yap hyperactivity in activated muscle stem (satellite) cells causes ERMS‐like tumours with high penetrance in mice 13. Whilst no *YAP1* or *WWTR1* point mutations have been reported for ERMS, mutations of several cancer genes that can crosstalk/interact with YAP or TAZ have been identified in sequencing studies 10. In addition, we and others have reported *YAP1* copy number gains in some rhabdomyosarcomas 13, 14.

Yap and Taz both have WW domains and can bind all Tead transcription factors 1, 2, but differ significantly in their function. This is especially evident in knockout mice, as a *Yap1* knockout is embryonal lethal at E8.5 15, whereas some *Wwtr1* knockout mice are born but later develop glomerulocystic kidney disease 16. Nonetheless, TAZ, like YAP, has been associated with cancer 17, 18, suggesting that both *YAP1* and *WWTR1* can act as oncogenes.

In the skeletal muscle lineage, high levels of YAP activity in muscle fibres cause myopathy 19, but more moderate increases induce skeletal muscle fibre hypertrophy 20, 21. In myoblasts and satellite cells, active Yap potently promotes proliferation 22, 23 and persistent YAP hyperactivity transforms satellite cells to cause ERMS 13. In contrast to Yap, active Taz has been reported to promote myogenic differentiation 24. The promotion of myogenic differentiation would be anti‐tumourigenic, because myoblasts within a rhabdomyosarcoma tumour fail to differentiate into post‐mitotic myocytes/myotubes 25. This might suggest context‐dependent function of TAZ as either an oncogene or tumour suppressor. Similarly, whilst YAP and TAZ generally function as oncogenes 17, 18, it has been reported that YAP can function as a tumour suppressor in the intestine 26, although there is no consensus on this 27.

The aim of this study was to test whether TAZ abundance is associated with survival in rhabdomyosarcoma and to characterize the cancer‐specific functions of TAZ in myoblasts and human ERMS cells, to identify TAZ as either an oncogene and YAP agonist or as a tumour suppressor.

## Materials and methods

### Human rhabdomyosarcoma tissue microarrays

For the rhabdomyosarcoma tissue microarrays, formalin‐fixed, paraffin‐embedded diagnostic tumour material from 79 patients with RMS was collected from UK centres through the Children's Cancer and Leukaemia Group (Local Research Ethics Committee Protocol Nos. 1836 and 2015 and Multi‐Regional Research Ethics Committee 06/4/71, with consent where required). The histology of cases was confirmed, by review according to World Health Organization guidelines, to be 25 alveolar and 54 embryonal. Cores of 0.6 mm diameter from three or more defined regions of tumour blocks were used to construct a tissue microarray 28. A previously described tissue microarray was also used, containing material from 60 alveolar and 171 embryonal cases 29. Immunohistochemistry and assessment of the arrays is reported in Supplementary materials and methods (see supplementary material).

### Cell culture

Mouse C2C12 and human RD and RH30 cells were cultured in Dulbecco's minimum essential medium (DMEM; Sigma), supplemented with 10% fetal calf serum (FCS; Hyclone). To induce differentiation, cells were cultured in growth medium until confluence, then the medium was switched to DMEM with 2% horse serum (Hyclone). Human cells 30 were cultured in skeletal muscle cell growth medium (Promocell, C‐23160) and passaged when needed.

### Retroviral and lentiviral expression vectors and transduction methods

Wild‐type TAZ and TAZ S89A cDNA were subcloned into a pMSCV–IRES–eGFP 31 retroviral expression backbone from plasmid DNA (Addgene Plasmids 24809 and 24815, deposited by Dr Jeff Wrana), creating pMSCV–3x Flag TAZ–IRES–eGFP and pMSCV–3x Flag–TAZ S89A–IRES–eGFP constructs. Empty vector pMSCV–IRES–eGFP was used as control vector. Retroviruses 23 and lentiviruses 32 were packaged in HEK293‐T cells, as described previously. Cells were transduced by incubation in diluted viral supernatant (1:4) until assayed.

### Immunocytochemistry

Fixed cells were permeabilized with 0.5% v/v Tween‐20/PBS for 6 min and blocked with 20% v/v goat serum/PBS for 30 min. The cells were incubated overnight at 4 °C with the primary antibodies mouse anti‐MyHC (DSHB, clone MF20; 1:400), mouse anti‐Ki67 (Cell Signaling, 9449; 1:400) and chicken anti‐GFP (Abcam, ab13970; 1:1000). Species‐specific, fluorochrome‐conjugated secondary antibodies were applied for 90 min at room temperature and the slides were mounted with Vectashield medium with DAPI (Vector laboratories). Proliferation of RD cells was assessed by EdU (5‐ethynyl‐2′‐deoxyuridine) incorporation, using 10 µm EdU for 4 h in growth medium. The Click‐iT® EdU kit (Invitrogen) was used to detect incorporated EdU, following the manufacturer's instructions. Quantification of Ki67‐ and EdU‐positive cell percentages and myogenic fusion index is reported in Supplementary materials and methods (see supplementary material).

### Western blotting

Cells were lysed in modified RIPA buffer supplemented with protease cocktail and phosphatase inhibitors (Sigma). Whole‐cell lysates were separated by SDS–PAGE electrophoresis and transferred to a nitrocellulose membrane. The membranes were then probed with mouse anti‐Yap/Taz (Santa Cruz, 101199; 1:100), rabbit anti‐phospho Yap Ser127 (Cell Signalling, 9411; 1:1000) or rabbit anti‐Taz (Sigma, HPA007415; 1:1000). Primary antibodies were then visualized using species‐specific conjugated secondary antibodies (Invitrogen) and digitally imaged.

### Anchorage‐independent agarose transformation assay

Cells (1 × 10^4^) were added to 2 ml growth medium with 0.5% agarose (Promega) and layered onto 2 ml 0.35% agarose‐supplemented medium in six‐well plates. The cells were fed with 1 ml growth medium weekly for 4–6 weeks, after which colonies were fixed with 10% acetic acid/10% methanol for 10 min, followed by staining with 0.005% crystal violet for 1 h, and counted using a light microscope. Colony numbers and sizes were determined using ImageJ v. 1.43 (NIH, USA).

### RNA extraction and reverse transcription (RT)–qPCR

Total RNA was isolated from cultured cells using Trizol reagent (Invitrogen). cDNA was synthesized by reverse transcription, using Superscript II (Invitrogen), and subjected to real‐time PCR with gene‐specific primers (see supplementary material, Table S1) in the presence of 1× Light Cycler 480® probes master mix (Roche). Relative abundance of mRNA was calculated by normalization to *Gapdh*, using mouse *Gapdh* endogenous control (Life Technologies, 4352339E) or human *GAPDH* (see supplementary material, Table S1).

### Luciferase reporter assays

For the luciferase reporter assay, retrovirally‐transduced C2C12 myoblasts or lentivirally‐transduced RD cells were seeded in six‐well plates overnight. Luciferase reporters (see supplementary material, Table S2) and TK–*Renilla* plasmid were co‐transfected and 24 h later cells were lysed and luciferase activity assayed using the Dual‐Luciferase Reporter Assay System (Promega), following the manufacturer's instructions. All luciferase activities were normalized to *Renilla* activity.

### Bioinformatic analyses

**Figure 1:**
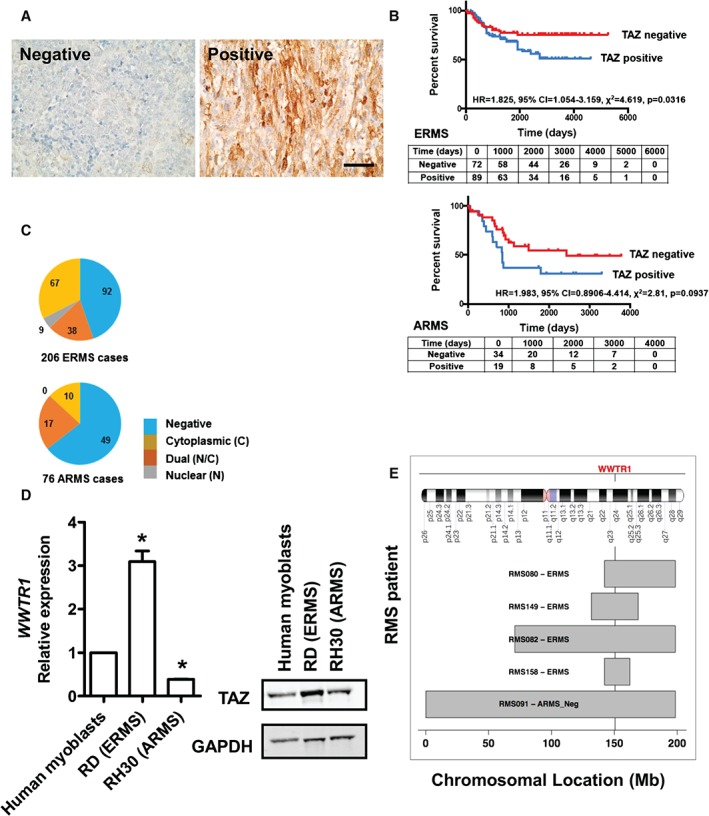
TAZ is associated with reduced survival in ERMS. (A) Examples of rhabdomyosarcoma, showing either negative or positive TAZ immunostaining; positive TAZ staining shows both strong nuclear and cytoplasmic TAZ staining, shown at higher magnification in Figure S1A (see supplementary material); scale bar = 50 µm. (B) Percentage survival of ERMS and ARMS patients who had sufficient follow‐up to be included in the survival analysis scored positive and negative for TAZ; the table shows patients at each given time point, ie those who had not died or had been lost to follow‐up. (C) Abundance and localization of TAZ in ERMS and ARMS. (D) (Left) Expression of WWTR1 and protein abundance in cultured human skeletal myoblasts, RD and RH30 cells; data are presented as mean ± SD, where n = 3 and * denotes a significant difference (p < 0.05), as assessed using one‐way ANOVA when compared to human myoblasts. (E) Genomic regions on chromosome 3 that include the WWTR1 locus were gained in five of 43 (12%) fusion‐negative rhabdomyosarcoma samples 33.

For Figure 1E, we determined copy number gains of the *WWTR1* locus (chromosome 3q24‐q24) by re‐analysing the Innovative Therapies for Children with Cancer/Carte d'Identite´ des Tumeurs (ITCC/CIT) dataset 33. For Figures S2A and S4B (see supplementary material) the gene expression profile of 235 rhabdomyosarcoma patients from two publicly available datasets were analysed: the first contained 101 samples (Innovative Therapies for Children with Cancer/Carte d'Identite des Tumeurs (ITCC/CIT) 33; the second 134 samples [Children's Oncology Group/Intergroup Rhabdomyosarcoma Study Group (COG/IRSG)] 34. Other non‐rhabdomyosarcoma datasets for these figures were as described in Tremblay *et al* 13. For Figures S2B and S4A (see supplementary material), the *Cancer Cell Line Encyclopaedia* 35 search engine was used (http://www.broadinstitute.org/ccle/home/26463/). For Figure S3A (see supplementary material), *WWTR1* and *YAP1* mutation and copy number data were plotted using cBioPortal (http://www.cbioportal.org/) 36, 37. For Figure S3B (see supplementary material), selected supplementary data from the dataset of Gentles *et al* 38 were used.

### Statistical analysis

Data were analysed using GraphPad Prism v. 5.0 (GraphPad Software) and were presented as mean ± standard deviation (SD). Statistical comparisons were done using one‐way ANOVA or unpaired *t*‐test. The Bonferroni multiple comparison test was applied following ANOVA to determine significant differences between the groups. For all experiments, statistical analysis was conducted on raw data collected from at least three independent experiments, performed on different occasions with three replicates each; *p* ≤ 0.05 was considered significant.

## Results

To test whether TAZ protein abundance is associated with clinical outcomes in rhabdomyosarcoma, we immunostained a human rhabdomyosarcoma tissue microarray containing 206 ERMS and 76 ARMS cases for TAZ, using the HPA007415 Sigma antibody, previously used to immunostain breast cancer samples 39, and assessed TAZ levels and location (Figure 1A, C). Nuclear staining for TAZ protein was observed in 22.8% (47/206) of ERMS samples (Figure 1C). Survival curves for TAZ‐positive and ‐negative samples were generated for tumours where data were available, and showed lower survival for TAZ‐positive rhabdomyosarcomas (Figure 1B). Survival was significantly lower for TAZ‐positive ERMS (*p =* 0.032) and there was also a trend for lower survival in TAZ‐positive ARMS (*p =* 0.094) when compared to TAZ‐negative ERMS and ARMS, respectively. Additionally, we measured *WWTR1* mRNA levels in human myoblasts 30, RD (ERMS) and RH30 (ARMS) cells 40 by RT–qPCR and also performed a western blot to compare TAZ protein levels in these cell lines. RD cells had the highest *WWTR1* mRNA and TAZ protein abundance, compared to RH30 cells and human skeletal myoblasts (Figure 1D). Re‐analysis of a previously publicly available dataset 33 revealed copy number gains of the region of Chromosome 3 that incorporates the *WWTR1* locus (chromosome 3q24‐q24) in 12% (five of 43) of human fusion gene‐negative rhabdomyosarcomas, but not in fusion gene‐positive ARMS (Figure 1E). Comparison with expression data showed that the expression level of the *WWTR1* gene correlates with the copy number of its chromosomal locus, with a Pearson correlation coefficient (*r*) *=* 0.31 for fusion gene‐negative rhabdomyosarcoma (*p =* 0.046).

Consistent with the association of TAZ immunostaining with ERMS rather than ARMS, *WWTR1* expression was also higher in ERMS than in PAX3/7–FOXO1‐positive ARMS and human skeletal muscle (see supplementary material, Figure S2A). To compare expression of *WWTR1* in RD and RH30 cells and soft tissue sarcomas generally in relation to other tissue cancer cell lines, we plotted their mRNA levels using the *Cancer Cell Line Encyclopedia* dataset (see supplementary material, Figure S2B) 35. This revealed that *WWTR1* and *YAP1* (not shown) were highly expressed in most cancer cell lines, with the exception of low expression in blood cancers. This analysis confirmed that *WWTR1* expression was high in ERMS cells consistent with higher expression in the RD (ERMS) than RH30 (ARMS) cell lines (Figure 1D).

We also plotted the pan‐cancer mutational profile for *WWTR1* and *YAP1* using cBioPortal 36, 37. Additionally, we displayed a meta‐*z* value for *WWTR1* and *YAP* expression as a measure of their association with survival in 18 000 cases of human cancer 38 (see supplementary material, Figure S3A, B). These analyses revealed few point mutations in *TAZ* and *YAP1*, but some copy number alterations were found (see supplementary material, Figure S3A). Across all cancers, expression of *TAZ* and *YAP1* was only moderately associated with poor survival (see supplementary material, Figure S3B) 38. *BIRC5* and *KLRB1* were also plotted as reference genes whose expression is most associated with poor and good survival, respectively (see supplementary material, Figure S3B).

**Figure 2:**
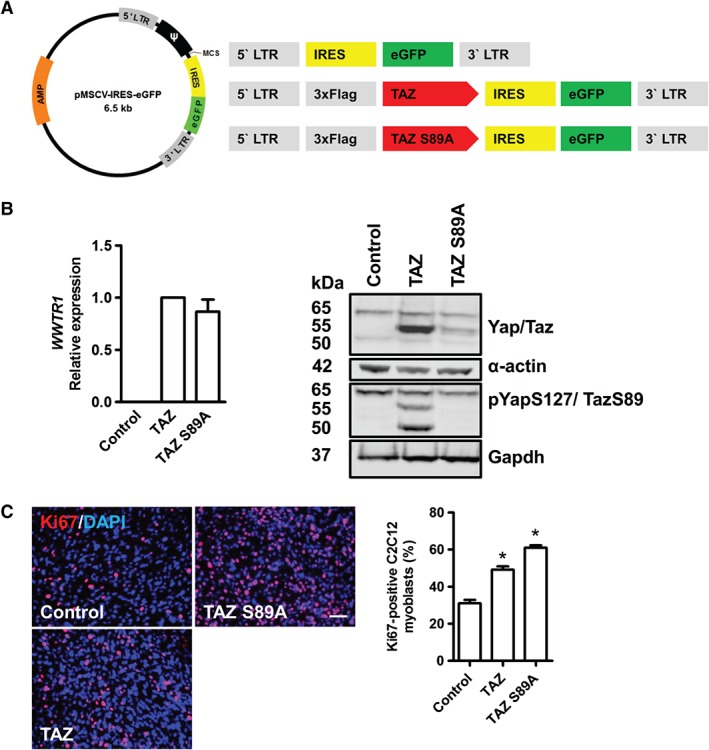
TAZ and TAZ S89A enhance proliferation in C2C12 myoblasts. (A) pMSCV map and schematic drawing of the control vector, TAZ and TAZ S89A‐encoding inserts. (B) Validation of human WWTR1 expression and TAZ protein levels in cells transduced with TAZ or TAZ S89A‐encoding retroviruses. (C) Example images of C2C12 myoblasts transduced with control vector, TAZ or TAZ S89A‐encoding retroviruses and immunostained 2 days later for the proliferation marker Ki67, and quantification of the proportion of Ki67‐expressing myoblasts; scale bar = 50 µm; data are expressed as mean ± SD, where n = 3 and * denotes a significant difference (p < 0.05), as assessed using one‐way ANOVA when compared to control.

The positive correlation between high *WWTR1* expression and poor survival prompted us to test whether expression of wild‐type TAZ or constitutively active TAZ S89A was sufficient to transform C2C12 mouse myoblasts. We subcloned the pMSCV retroviral backbone (containing an internal ribosomal entry site to allow parallel expression of eGFP) with flag‐tagged human TAZ and TAZ S89A, with pMSCV–IRES–eGFP serving as a control (Figure 2A). Retroviral constructs were verified by RT–qPCR, where expression of human *WWTR1* mRNA was detected from both the TAZ and TAZ S89A‐encoding retroviruses (Figure 2B). Western blot analysis of transduced cells also confirmed the generation of wild‐type TAZ and constitutively active TAZ S89A from the retroviral constructs (Figure 2B). The over‐expression of TAZ mutants in C2C12 myoblasts or knockdown of TAZ in RD cells did not affect YAP abundance (Figures 2B, 5A).

To measure the proliferation of C2C12 myoblasts, we transduced myoblasts with the TAZ or TAZ S89A‐encoding retroviruses and immunostained for the proliferation marker Ki67. Expression of TAZ significantly increased the proportion of Ki67‐positive cells by 59% and TAZ S89A increased it by 96%, compared to control vector (Figure 2C), revealing that enhanced TAZ levels and activity promotes proliferation in skeletal myoblasts.

**Figure 3:**
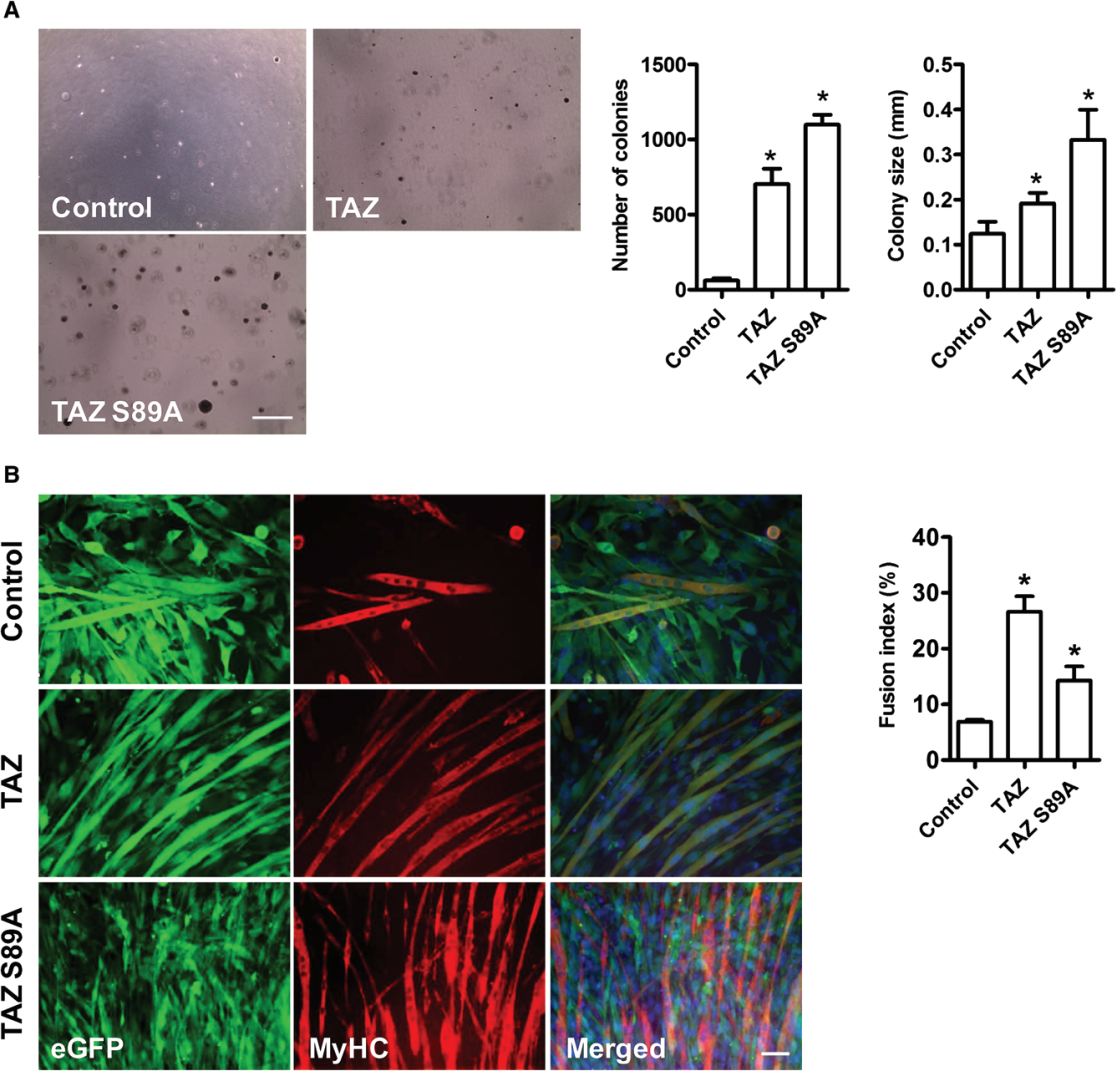
TAZ or mutant TAZ S89A promote transformation of C2C12 myoblasts. (A) Example images showing colony formation in C2C12 myoblasts transduced with empty vector, TAZ or TAZ S89A‐encoding retroviruses; TAZ or TAZ S89A significantly increase colony number and size when compared to control vector. (B) Expression of TAZ or TAZ S89A did not prevent myogenic differentiation of C2C12 myoblasts when compared to control (empty vector). TAZ or TAZ S89A, actually significantly increased the fusion index after 72 h. Data are expressed as mean ± SD, where n = 3 and * indicates a significant difference (p < 0.05) using one‐way ANOVA when compared to control; scale bar = (A) 500 µm; (B) 50 µm

Next, we tested the effects of TAZ or TAZ S89A expression on C2C12 myoblast colony formation on soft agar, as a measure of transformation. TAZ expression significantly increased the mean number of colonies by 12‐fold and TAZ S89A by 18‐fold when compared to control vector (Figure 3A). These colonies were also bigger in size with TAZ or TAZ S89A expression (Figure 3A). TAZ or TAZ S89A expression did not impair myogenic differentiation in immortalized C2C12 myoblasts (Figure 3B), consistent with earlier findings 22, and in marked contrast to the inhibitory effects of YAP1 S127A on differentiation 24.

**Figure 4:**
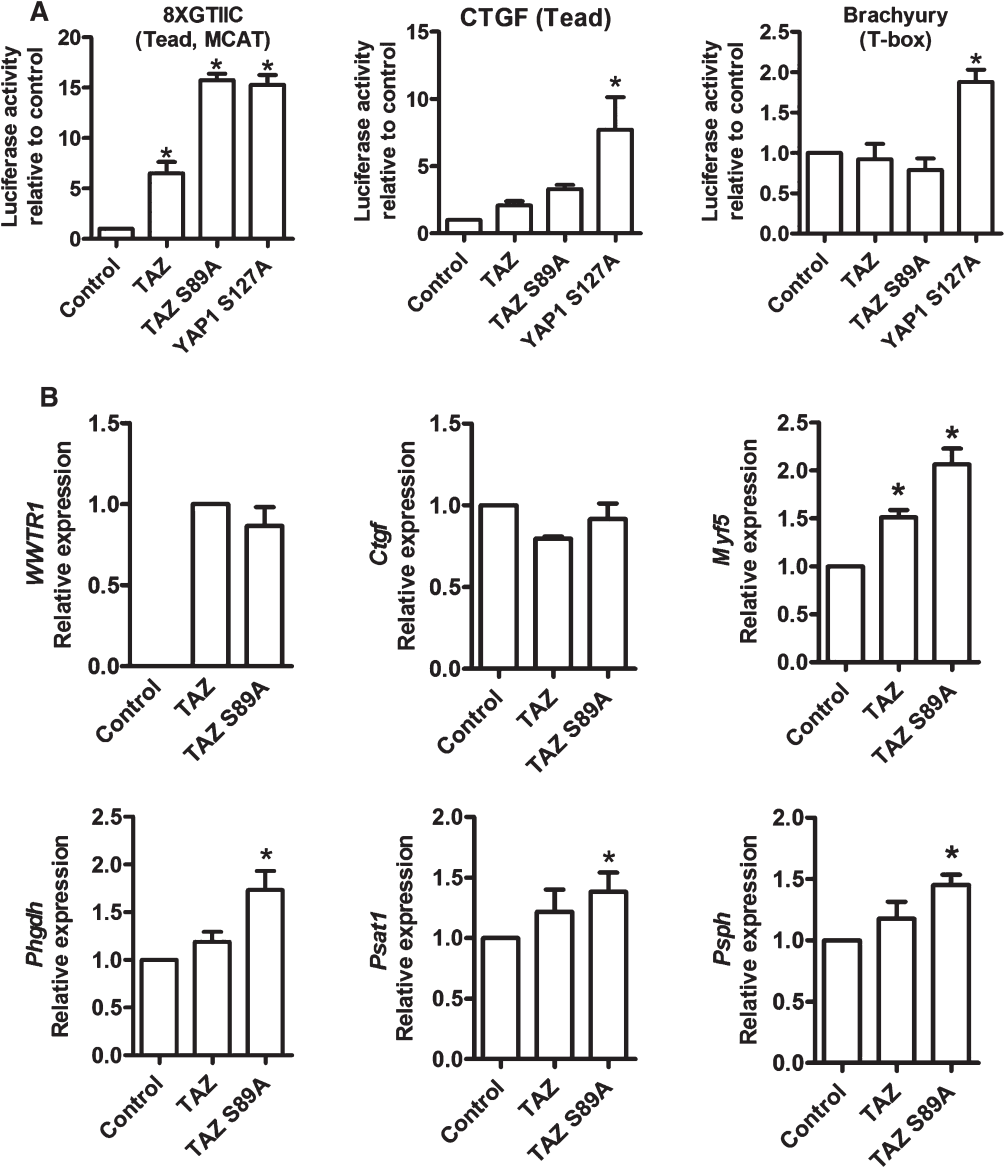
TAZ S89A activates genes associated with human cancer. (A) Effects of TAZ, mutant TAZ S89A and YAP S127A expression on Hippo pathway reporter constructs 8XGTIIC–Luc, CTGF–Luc and Brachyury–Luc in C2C12 myoblasts. (B) Validation of the expression of human WWTR1 and effects of TAZ or TAZ S89A expression in C2C12 myoblasts on expression of Ctgf, Myf5, Phgdh, Psat1 and Psph. Data are expressed as mean ± SD, where n = 3 and * indicates a significant difference (p < 0.05) when compared to control using one‐way ANOVA

To test whether the different functions of TAZ and YAP can be explained by different transcriptional activities, we used a panel of Hippo signalling reporters (see supplementary material, Table S2), including the 8XGTIIC Hippo 41, 42, CTGF (connective tissue growth factor; a Hippo reporter with three Tead binding sites from this gene 32) and a Brachyury reporter (as YAP has been reported to bind TBX5 in tumour cell lines 8). C2C12 myoblasts were infected with retroviral vectors encoding wild‐type TAZ, TAZ S89A or YAP1 S127A mutants and then transfected with reporter constructs. Measuring normalized luciferase activity from the reporter constructs revealed that while mutant TAZ S89A or YAP1 S127A activated the 8XGTIIC reporter by similar amounts, only YAP1 S127A significantly enhanced the activity of the CTGF and Brachyury reporters (Figure 4A).

Next, we searched for transcriptional mechanisms by which TAZ S89A drives tumour‐related changes in myoblasts. To do this we retrovirally expressed TAZ or TAZ S89A and used RT–qPCR to measure expression of the YAP activity markers *Ctgf*, the zebrafish embryonal cancer stem cell gene *Myf5* 43 and *Phgdh*, *Psat1* and *Psph* of the serine biosynthesis pathway, previously linked to remodelling of metabolism in cancer 44, 45 and induced by Yap in myoblasts 23 and Yap‐driven ERMS 13. TAZ S89A drove increased expression of the cancer‐related genes *Phgdh*, *Psat1*, *Psph* and *Myf5*, although *Ctgf* mRNA levels were unchanged (Figure 4B). This effect of TAZ on *Myf5* in C2C12 myoblasts is consistent with the 8.8‐fold up‐regulation in YAP1 S127A‐driven ERMS 13 and high *MYF5* expression in the RH18 (ERMS) cell line and in human ERMS compared to skeletal muscle or human ARMS (see supplementary material, Figure S4). Thus, constitutive active TAZ can drive expression of key rhabdomyosarcoma and cancer‐related genes.

**Figure 5:**
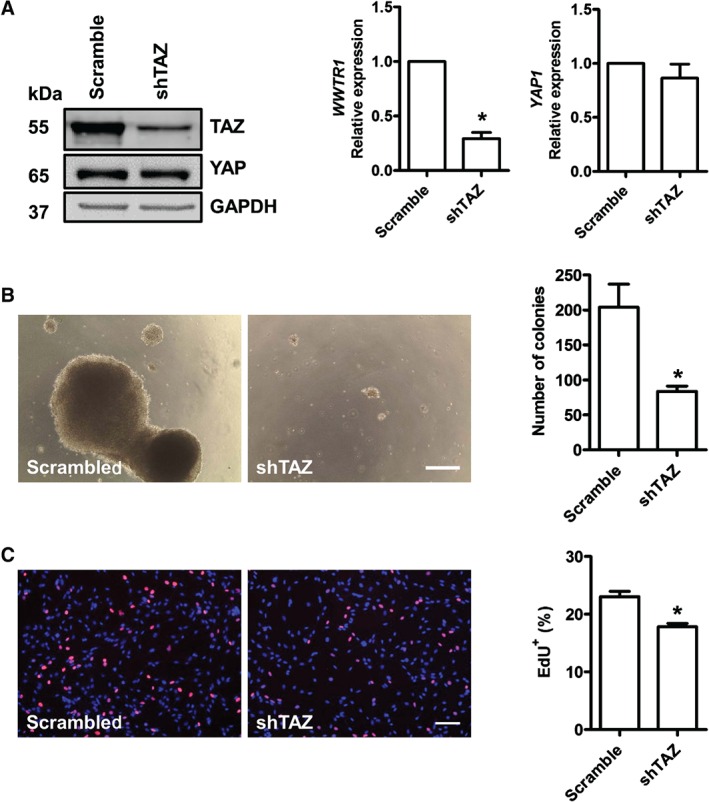
TAZ knockdown reduces proliferation and transformation behaviour in RD cells. (A) Transduction of RD cells with scrambled or TAZ shRNA‐encoding lentiviruses for 24 h followed by 72 h puromycin selection reduced TAZ protein and mRNA but did not affect YAP expression, as assessed by western blot and qPCR. (B) Example images showing number of colonies and their size with RD cells transduced with either scrambled or TAZ shRNA; TAZ knockdown significantly reduced colony number when compared to control. (C) Example images of RD cells transduced with scrambled or TAZ shRNA lentiviruses and incorporation of EdU visualized and quantified. Data are expressed as mean ± SD, where n = 3 and * denotes a significant difference (p < 0.05) when compared to control using unpaired t‐test; scale bar = (B) 500 µm; (C) 50 µm

**Figure 6:**
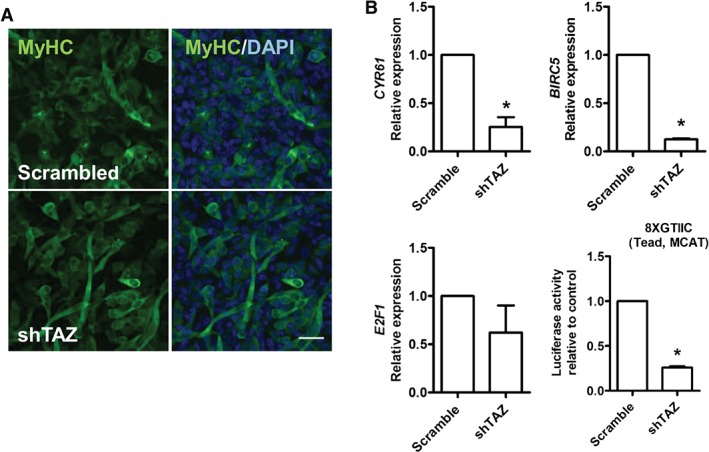
TAZ knockdown reduces the levels of known oncogenes in RD cells. (A) shRNA‐mediated knockdown of TAZ in RD cells had no effect on myogenic differentiation, as shown by immunostaining with MyHC. (B) Expression of CYR61, E2F1, BIRC5 and activity of the 8XGTIIC–Luc reporter was assessed in RD cells after TAZ knockdown. Data are expressed as mean ± SD, where n = 3 and * indicates a significant difference (p < 0.05) when compared to control using unpaired t‐test; scale bar = 50 µm

To test whether TAZ is critical for the tumour characteriztics of RD cells, where TAZ protein levels are high (Figure 1D), we investigated the effects of lentiviral shRNA‐mediated TAZ knockdown (see supplementary material, Table S3) on the proliferation and differentiation of RD cells. As expected, RD cells transduced with *TAZ* shRNA‐encoding lentiviruses had less TAZ protein and expressed lower levels of *TAZ* mRNA than control cells transduced with scrambled shRNA sequences. Again, manipulation of TAZ protein levels did not alter *YAP1* mRNA or YAP protein levels (Figure 5A). *WWTR1* shRNA‐transduced RD cells cultured in soft agar formed significantly fewer colonies when compared to control cells transduced with scrambled shRNA sequences, which were also smaller (Figures 5B). TAZ knockdown in RD cells also reduced the proliferation rate, as measured by EdU incorporation (Figure 5C). Immunostaining for MyHC after TAZ knockdown in RD cells revealed that reduction of TAZ levels did not affect myogenic differentiation (Figure 6A).

Next, we performed qPCR analyses to assess the effect of TAZ knockdown in RD cells on expression of some known Hippo target genes. TAZ knockdown significantly reduced the expression of *CYR61* by ∼75% and *BIRC5* (survivin) by ∼90% when compared to controls (Figure 6B). There was also a trend for reduced *E2F1* expression after TAZ knockdown, but this did not reach significance (*p =* 0.06) (Figure 6B). Furthermore, TAZ knockdown in RD cells significantly reduced the transcriptional activity of the 8XGTIIC (TEAD) luciferase reporter by ∼70% (Figure 6B), suggesting that TEADs are key targets of TAZ in RD cells. Together, these data further indicate that *TAZ* acts as an oncogene in RD cells.

## Discussion

Our data suggest that TAZ functions as an oncogene in ERMS. However, whilst YAP1 S127A expression blocks myogenic differentiation, we confirm that TAZ S89A expression does not inhibit differentiation, in line with an earlier study 24. Consistent with its oncogene function, TAZ drives expression of the ERMS stem cell factor *Myf5* 43 and of members of the serine biosynthesis pathway (*Phgdh*, *Psat1* and *Psph*), which have been linked to metabolic remodelling in cancer 44, 45. Moreover, knockdown of TAZ in human ERMS RD cells reduces both proliferation and anchorage‐independent growth.

*WWTR1* mRNA is more highly expressed in ERMS than in *PAX3/7–FOXO1*A‐positive ARMS and differentiated skeletal muscle. Similarly, TAZ protein is detected in 55% of human ERMS but only in 36% of human ARMS cases. These observations, and our previous results that identified more YAP immunostaining in ERMS than ARMS, suggest that YAP and TAZ are generally more abundant in ERMS than in ARMS. Copy number gains can explain the higher abundance of YAP in some ERMS cases 13, 14 and here we additionally report copy number gains of the *WWTR1* locus in 12% of ERMS and fusion gene‐negative ARMS cases, but not in any ARMS case 46.

We previously reported that YAP abundance and activity are associated with higher grades and reduced survival in ERMS 13. Here we show that the abundance of TAZ protein is similarly associated with reduced survival in ERMS (*p =* 0.032) and that there is a trend for ARMS (*p =* 0.094), suggesting that TAZ is functionally important, especially in ERMS. Similar associations between TAZ abundance and poor survival have been reported for human breast cancer 39, non‐small cell lung cancer 47, hepatocellular carcinoma 48 and colorectal cancer 49. In breast cancer, higher Hippo activity was detected in higher‐grade breast cancers and in this cancer TAZ was also linked to cancer stem cells and drug resistance 39.

Rhabdomyosarcomas comprise cells/myoblast‐like cells that fail to differentiate into post‐mitotic, multinucleated myotubes and fully mature muscle fibres 25. Intriguingly, TAZ expression increases during myogenic differentiation of human fetal myoblasts and TAZ has been reported to promote myogenic differentiation 24, whereas YAP inhibits differentiation 20, 23. In our experiments, TAZ S89A and TAZ expression increased proliferation and soft agar growth of C2C12 myoblasts, whereas TAZ knockdown in RD cells reduced proliferation, soft agar growth, activity of the 8XGTIIC reporter and expression of genes such as *CYR61* and *BIRC5*. Collectively, this suggests that TAZ acts, like YAP 13, as an oncogene in the muscle lineage.

Intriguingly, whilst YAP1 S127A (but not YAP) prevents differentiation of C2C12 myoblasts 22, TAZ S89A and TAZ enhanced myogenic differentiation into multinucleated, myosin heavy chain‐positive myotubes. This context‐dependent, seemingly contradictory pro‐proliferation and pro‐differentiation function of TAZ is reminiscent of the function of Notch in the muscle lineage, as Notch can affect both the quiescence and proliferation of muscle stem cells, depending on the context and timing 50.

To identify potential mechanisms by which TAZ exerts its effects, we conducted reporter and gene expression experiments. TAZ and YAP mainly bind TEAD transcription factors that regulate gene expression through binding to CATTCC/GGAATG motifs (MCAT; TEADs bind both strands 1, 2). Additionally, YAP and TAZ have been reported to bind other transcription factors, including TBX5 8, 51, which has been identified as an important interaction in cancer 8. To identify putative mechanisms for the different actions of YAP and TAZ, we investigated the effect of TAZ and YAP mutants on 8XGTIIC, CTGF and Brachyury (T‐box) reporters. This revealed that, whilst TAZ S89A and YAP1 S127A similarly activated the 8XGTIIC reporter 41, 42, only YAP1 S127A was able to significantly activate the CTGF 32 and Brachyury reporters. This suggests that TAZ and YAP have common and unique effects on transcription, which could explain the difference in function seen in skeletal muscle and in *Yap1* 15 and *Wwtr1* knockout mice 16.

Finally, we determined the effect of wild‐type TAZ or TAZ S89A expression on genes that we had previously identified as Yap‐regulated genes in myoblasts 23 and Yap‐driven ERMS 13.

CTGF is commonly used as a marker of Hippo activity, as it has three MCAT Tead‐binding sites in its proximal promoter 32. We found no effect of TAZ or TAZ S89A expression on *Ctgf* expression. In contrast to this, YAP S127A increases *Ctgf* expression in myoblasts 23 but *Ctgf* expression is not increased in Yap‐driven ERMS 13. This suggests that *Ctgf* expression is not a universal marker of high YAP/TAZ‐Tead activity.

We found that TAZ and TAZ S89A increased *Myf5* expression. This is functionally relevant, as *Myf5* is a cancer stem cell gene in zebrafish ERMS 43. Intriguingly, the ECR111 enhancer of *Myf5* harbours a Tead‐targeted MCAT motif 52 which could act as a target for TAZ. Previously we found that especially *YAP1 S127A* increased *Myf5* expression in C2C12 myoblasts and Yap‐ERMS 13. Together this suggests that YAP and TAZ may contribute to the maintenance of a cancer stem cell population in ERMS through the expression of *Myf5*.

Cancer cells remodel their metabolism so that glycolytic intermediates and other metabolites are channelled into biosynthetic pathways to support growth and proliferation. This is known as aerobic glycolysis or the Warburg effect 53. The serine biosynthesis pathway, and especially the first enzyme of this pathway, *Phgdh*, have previously been identified as proliferation‐limiting genes in cancer, and some copy number gains of *PHGDH* have been identified in some cancers 44, 45. We found that TAZ S89A significantly increased expression of *Phgdh*, *Psat1* and *Psph*, the three enzymes of the serine biosynthesis pathway. The TAZ‐driven induction of the serine biosynthesis pathway is a novel mechanism of metabolic remodelling and complements previous studies that linked Hippo signalling to the Warburg effect in cancer 54, 55.

Interestingly, YAP has recently been shown to inversely regulate TAZ protein, but this inverse relationship is unidirectional, only being observed upon modulation of YAP and not TAZ 56, This inverse relationship extends to skeletal muscle, since overexpression of TAZ mutants in C2C12 myoblasts or knockdown of TAZ in RD cells do not seem to have compensatory effects on YAP in RD cells.

In summary, our data identify TAZ as an oncogene in the muscle lineage. This further supports an important role for the Hippo pathway in both ERMS 13, ARMS 57 and sarcoma in general 58, 59.

## Author contributions

Abdalla Mohamed performed most, and Congshan Sun and Vanessa De Mello contributed to, the cell culture experiments. Jonanna Selfe and Janet Shipley provided the tissue microarrays which were immunostained and scored by Abdalla Mohamed and Graeme I Murray and statistically analysed by Jonanna Selfe and Janet Shipley. Edoardo Missiaglia carried out bioinformatic analyses. Abdalla Mohamed and Henning Wackerhage wrote the manuscript and Peter S Zammit and Henning Wackerhage planned the study and obtained funding.

**SUPPLEMENTARY MATERIAL ONLINE**

**Supplementary materials and methods**

**Supplementary figure legends**

**Figure S1.** Examples of immunohistochemistry

**Figure S2.** Analyses of levels of *WWRT1* and *YAP1*

**Figure S3.** Mutations in *WWTR1* and *YAP1*, and survival analyses

**Figure S4.** Analyses of *Myf5* expression in cancer cell lines and of *MYF5* in different cohorts

**Table S1.** RT–qPCR primers used in this study

**Table S2.** Name, description and ratio (firefly:*Renilla*) of the luciferase constructs used in dual‐luciferase reporter assays

**Table S3.** Catalogue number and the target shRNA sequences of scramble control shRNA and TAZ shRNA

## Supporting information


**Supplementary materials and methods**

**Supplementary figure legends**

**Figure S1.** Examples of immunohistochemistry. (A) Higher magnification for strong positive TAZ staining with nuclear and cytoplasmic localization. (B) Example images of C2C12 myoblasts transduced with different constructs grown at high confluence and immunostained for the proliferation markers Ki67 and eGFP; scale bars (A, B) = 50 µm

**Figure S2.** Analyses of levels of *WWRT1* and *YAP1*. (A) *WWTR1* expression is higher in ERMS than in PAX3/7‐FOXO1‐positive ARMS and skeletal muscle in both the ITCC/CIT and COG/IRSG datasets; *WWTR1* expression increases during myogenic differentiation (FetMyob‐1–3); NB, neuroblastoma; ES, Ewing syndrome; WT, Wilms' tumour; MBL, medulloblastoma; these were used as small round tumours for comparison. (B) *WWTR1* and *YAP1* are highly expressed in soft tissue, including rhabdomyosarcoma cancer cell lines, when especially compared to blood cancers; the data were obtained from the *Cancer Cell Line Encyclopedia* (CCLE) [35]; specifically, the log2 expression of *WWTR1* in the RD (ERMS) cells was 10.5 and in the RH30 (ARMS) cells was 8.8

**Figure S3.** Mutations in *WWTR1* and *YAP1*, and survival analyses. (A) *WWTR1* and *YAP1* mutations in 24293 human tumour samples. (B) The association between *WWTR1* and *YAP1* expression and poor survival in 18 000 cases of cancer; *BIRC5* and *KLRB1* are also shown as genes whose expression is most and least associated with poor survival in human cancer, respectively

**Figure S4.** Analyses of *Myf5* expression in cancer cell lines and of *MYF5* in different cohorts. (A) *Myf5* expression in cancer cell lines; note the high‐level expression of Myf5 in the RH18 (ERMS) and RH30 (ARMS) cell lines. (B) MYF5 expression in human skeletal (sk.) muscle, ERMS, fusion gene‐negative ARMS (ARMS_Neg) and *PAX3/7‐FOXO1*‐positive ARMS (ARMS_Pax3, ARMS_Pax7) in the ITCC/CIT [33] and COG/IRSG cohorts [34]

**Table S1.** RT–qPCR primers used in this study
**Table S2.** Name, description and ratio (firefly:Renilla) of the luciferase constructs used in dual‐luciferase reporter assays
**Table S3.** Catalogue number and the target shRNA sequences of scramble control shRNA and TAZ shRNA
