# Supplementary material for: The Hippo effector TAZ (WWTR1) transforms myoblasts and TAZ abundance is associated with reduced survival in embryonal rhabdomyosarcoma
Source: J Pathol. 2016 Aug 22;240(1):3–14. doi: 10.1002/path.4745 (PMC4995731; doi:10.1002/path.4745)
Supplement: Supplementary file 1 — Supplementary materials and methods [file PATH-240-3-s004.doc]

+A: **Supplementary materials and methods**

+B: Immunohistochemistry of RMS tissue array

Formalin-fixed, paraffin-embedded sections were dewaxed in xylene, rehydrated in alcohol and an antigen retrieval step performed when required. This step consisted of microwaving the sections fully immersed in 10 mm citrate buffer at pH 6.0 for 20 min in an 800 W microwave oven operated at full power. The sections were then allowed to cool to room temperature. Primary anti-Taz antibody (1 in 100; Sigma, HPA007415) was applied for 60 min at room temperature, washed with buffer (Dako) with subsequent peroxidase blocking for 5 min (Dako). This was followed by a single 2 min buffer wash, after which prediluted peroxidase polymer-labelled goat anti-mouse/rabbit secondary antibody (Envision™, Dako) was applied for 30 min at room temperature, followed by further washing with buffer to remove unbound antibody. Sites of peroxidase activity were then demonstrated, with diaminobenzidine as the chromogen applied for three successive 5 min periods. Finally, the sections were washed in water, lightly counterstained with haematoxylin, dehydrated and mounted. Omitting the primary antibody from the immunohistochemical procedure and replacing it with antibody diluent acted as a negative control.

+B: Assessment of immunohistochemistry

Following the completion of the immunohistochemistry, the tissue microarray cores were examined by light microscopy and the intensity of immunostaining was quantified using a semi-quantitative scoring method. The subcellular localization (nuclear, cytoplasmic) of TAZ immunoreactivity was also noted. The scoring was performed by GIM, a pathologist. An overall 'positive' staining refers to any staining, whether it was in the nucleus, cytoplasm or both, versus 'negative' for no staining at all.

+B: Quantification of Ki67^+^ and EdU^+^ cell percentage and fusion index percentages

For plated cells, counting of Ki67- or EdU-positive cells and calculating myogenic fusion index were conducted on a minimum of three representative images (random fields of view)/replicate, using the cell counter plugin of ImageJ v. 1.43 (NIH, USA). For calculating the proportion of Ki67- and EdU-positive cells, the number of Ki67- and EdU-positive cells was divided by the total number of nuclei within each field. For the myogenic fusion index, the number of nuclei in MyHC-positive myotubes was counted and divided by the total number of nuclei within each field.
