# Supplementary material for: The Hippo effector TAZ (WWTR1) transforms myoblasts and TAZ abundance is associated with reduced survival in embryonal rhabdomyosarcoma
Source: J Pathol. 2016 Aug 22;240(1):3–14. doi: 10.1002/path.4745 (PMC4995731; doi:10.1002/path.4745)
Supplement: Supplementary file 2 — Supplementary figure legends [file PATH-240-3-s005.doc]

**<Supplementary material>**

**Figure S1.** Examples of immunohistochemistry. (A) Higher magnification for strong positive TAZ staining with nuclear and cytoplasmic localization. (B) Example images of C2C12 myoblasts transduced with different constructs grown at high confluence and immunostained for the proliferation markers Ki67 and eGFP; scale bars (A, B) = 50 µm

**Figure S2.** Analyses of levels of *WWRT1* and *YAP1*. (A) *WWTR1* expression is higher in ERMS than in PAX3/7–FOXO1-positive ARMS and skeletal muscle in both the ITCC/CIT and COG/IRSG datasets; *WWTR1* expression increases during myogenic differentiation (FetMyob-1–3); NB, neuroblastoma; ES, Ewing syndrome; WT, Wilms’ tumour; MBL, medulloblastoma; these were used as small round tumours for comparison. (B) *WWTR1* and *YAP1* are highly expressed in soft tissue, including rhabdomyosarcoma cancer cell lines, when especially compared to blood cancers; the data were obtained from the *Cancer Cell Line Encyclopedia* (CCLE) [35]; specifically, the log2 expression of *WWTR1* in the RD (ERMS) cells was 10.5 and in the RH30 (ARMS) cells was 8.8

**Figure S3.** Mutations in *WWTR1* and *YAP1*, and survival analyses. (A) *WWTR1* and *YAP1* mutations in 24293 human tumour samples. (B) The association between *WWTR1* and *YAP1* expression and poor survival in 18 000 cases of cancer; *BIRC5* and *KLRB1* are also shown as genes whose expression is most and least associated with poor survival in human cancer, respectively

**Figure S4.** Analyses of *Myf5* expression in cancer cell lines and of *MYF5* in different cohorts. (A) *Myf5* expression in cancer cell lines; note the high-level expression of *Myf5* in the RH18 (ERMS) and RH30 (ARMS) cell lines. (B) MYF5 expression in human skeletal (sk.) muscle, ERMS, fusion gene-negative ARMS (ARMS_Neg) and *PAX3/7–FOXO1*-positive ARMS (ARMS_Pax3, ARMS_Pax7) in the ITCC/CIT [33] and COG/IRSG cohorts [34]
