# Supplementary figures and images for: The Hippo effector TAZ (WWTR1) transforms myoblasts and TAZ abundance is associated with reduced survival in embryonal rhabdomyosarcoma
Source: J Pathol. 2016 Aug 22;240(1):3–14. doi: 10.1002/path.4745 (PMC4995731; doi:10.1002/path.4745)

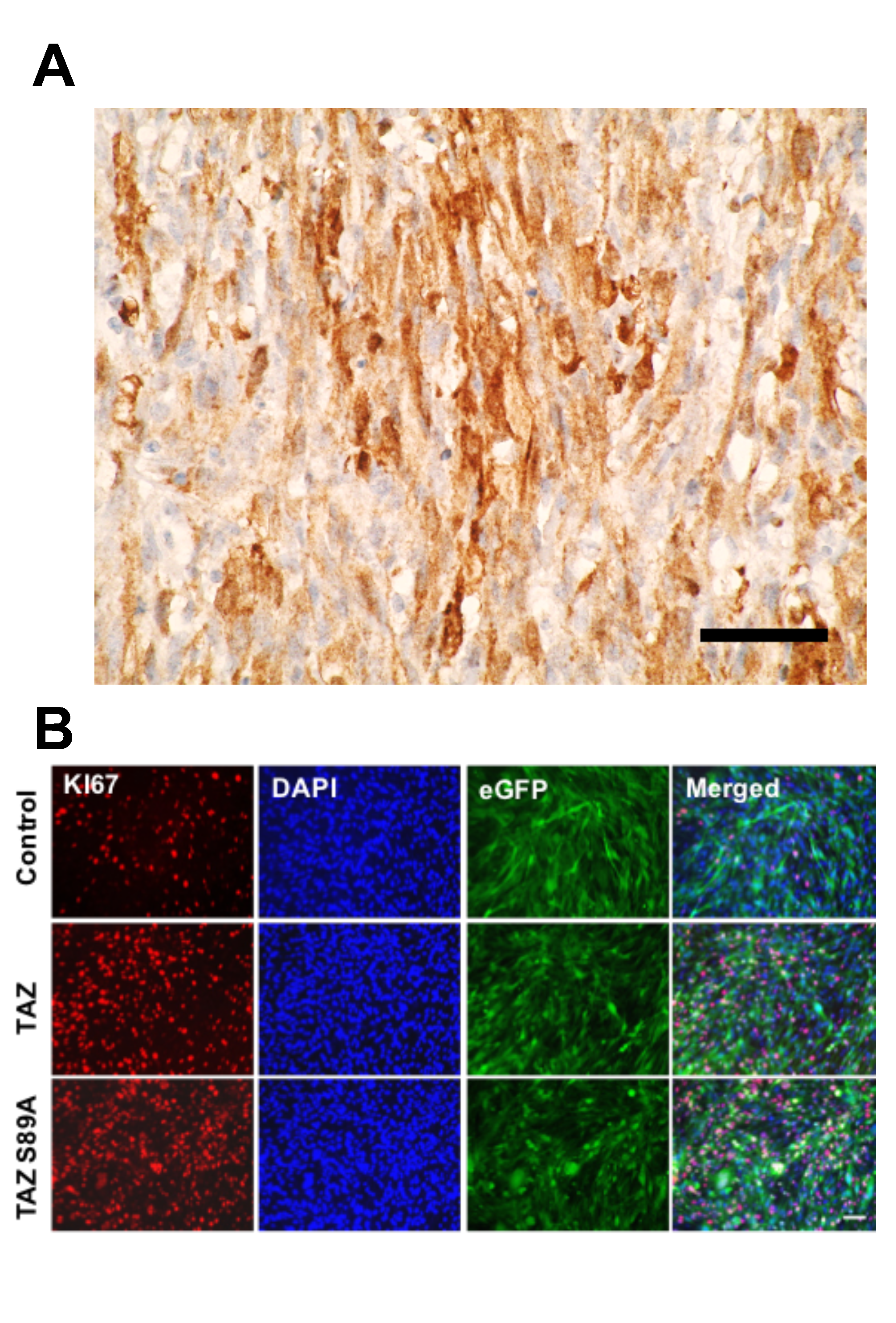

Supplement: Supplementary file 3 — Figure S1. Examples of immunohistochemistry. (A) Higher magnification for strong positive TAZ staining with nuclear and cytoplasmic localization. (B) Example images of C2C12 myoblasts transduced with different constructs grown at high confluence and immunostained for the proliferation markers Ki67 and eGFP; scale bars (A, B) = 50 µm [file PATH-240-3-s002.tif]

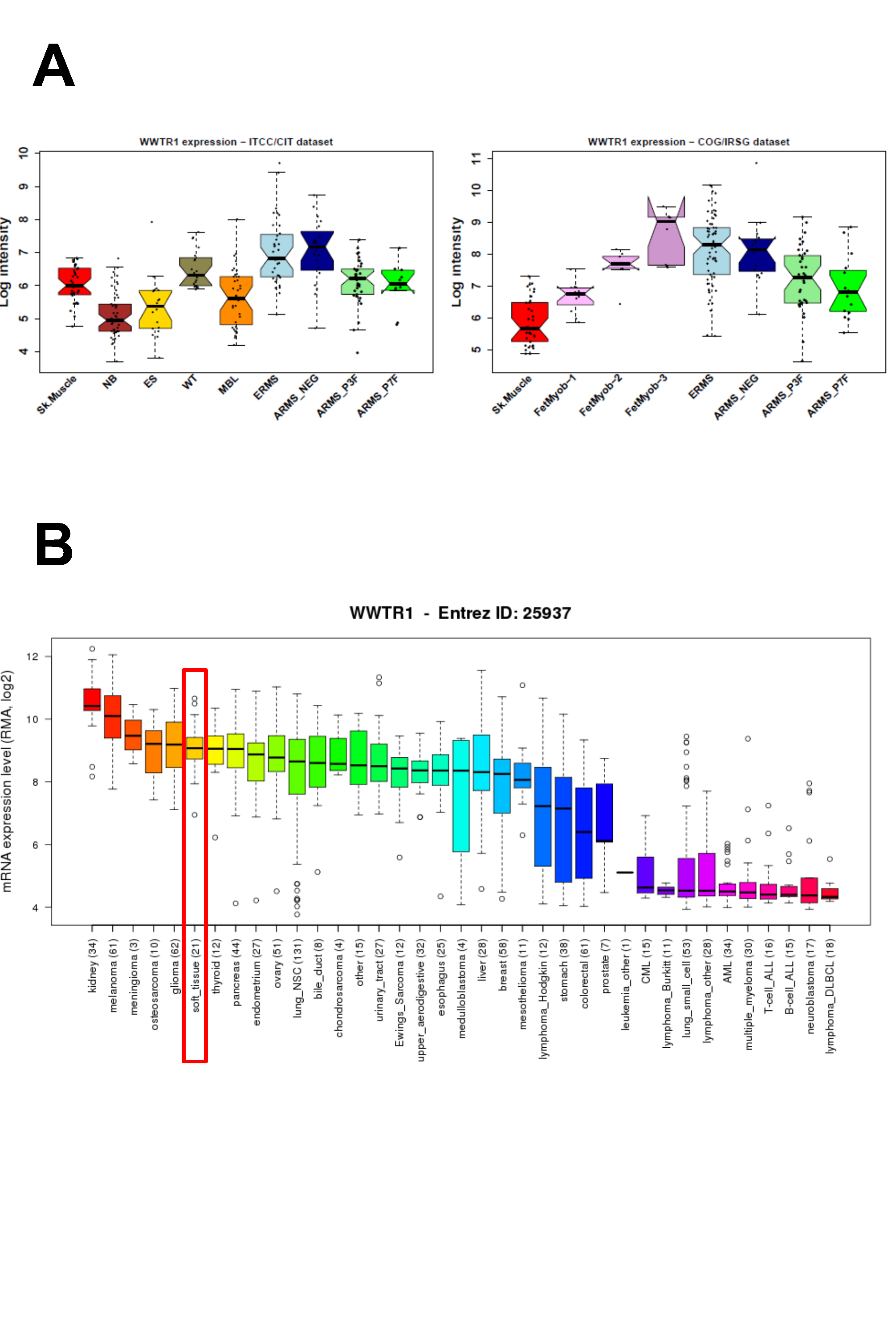

Supplement: Supplementary file 4 — Figure S2. Analyses of levels of WWRT1 and YAP1. (A) WWTR1 expression is higher in ERMS than in PAX3/7‐FOXO1‐positive ARMS and skeletal muscle in both the ITCC/CIT and COG/IRSG datasets; WWTR1 expression increases during myogenic differentiation (FetMyob‐1–3); NB, neuroblastoma; ES, Ewing syndrome; WT, Wilms' tumour; MBL, medulloblastoma; these were used as small round tumours for comparison. (B) WWTR1 and YAP1 are highly expressed in soft tissue, including rhabdomyosarcoma cancer cell lines, when especially compared to blood cancers; the data were obtained from the Cancer Cell Line Encyclopedia (CCLE) [35]; specifically, the log2 expression of WWTR1 in the RD (ERMS) cells was 10.5 and in the RH30 (ARMS) cells was 8.8 [file PATH-240-3-s001.tif]

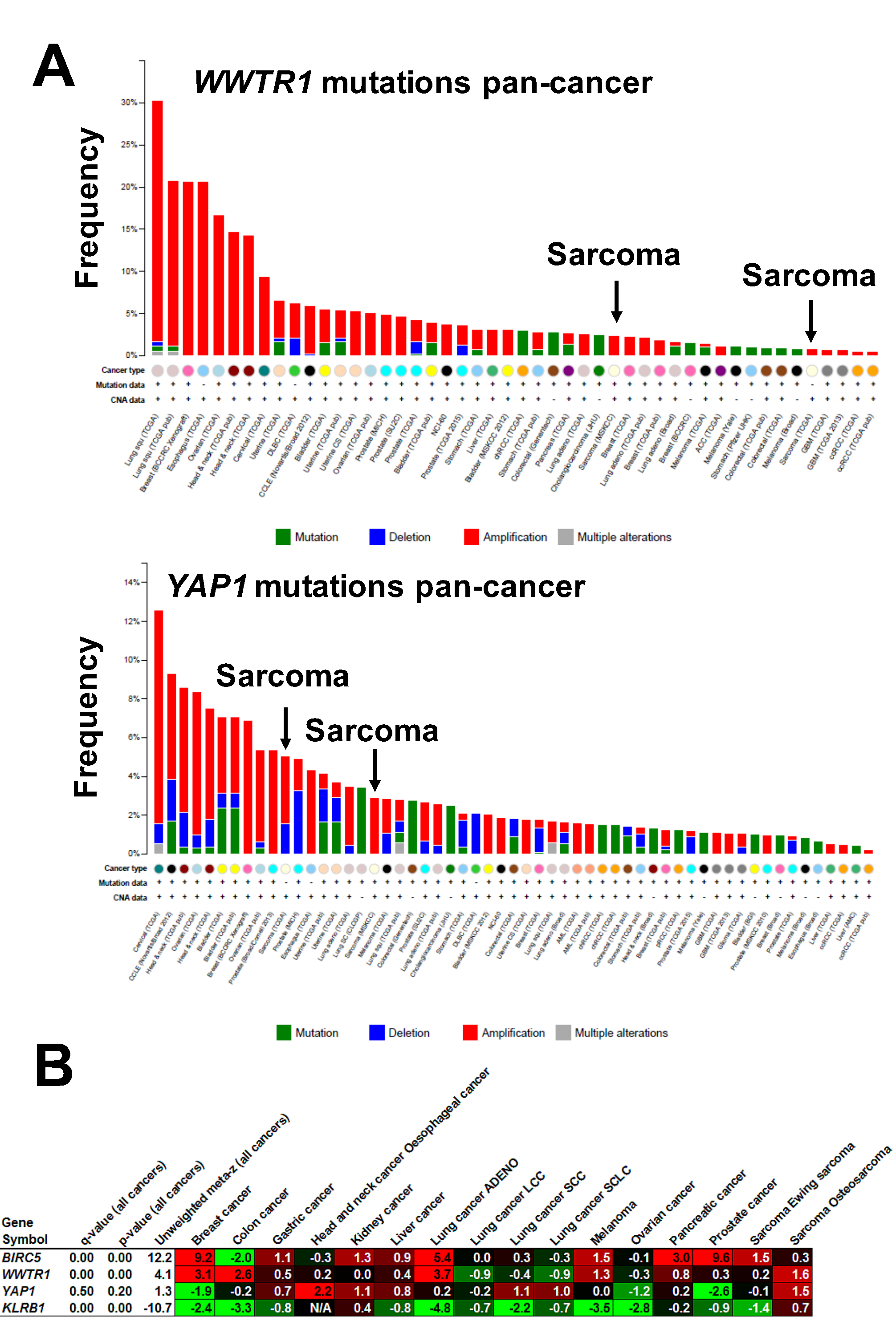

Supplement: Supplementary file 5 — Figure S3. Mutations in WWTR1 and YAP1, and survival analyses. (A) WWTR1 and YAP1 mutations in 24293 human tumour samples. (B) The association between WWTR1 and YAP1 expression and poor survival in 18 000 cases of cancer; BIRC5 and KLRB1 are also shown as genes whose expression is most and least associated with poor survival in human cancer, respectively [file PATH-240-3-s003.tif]

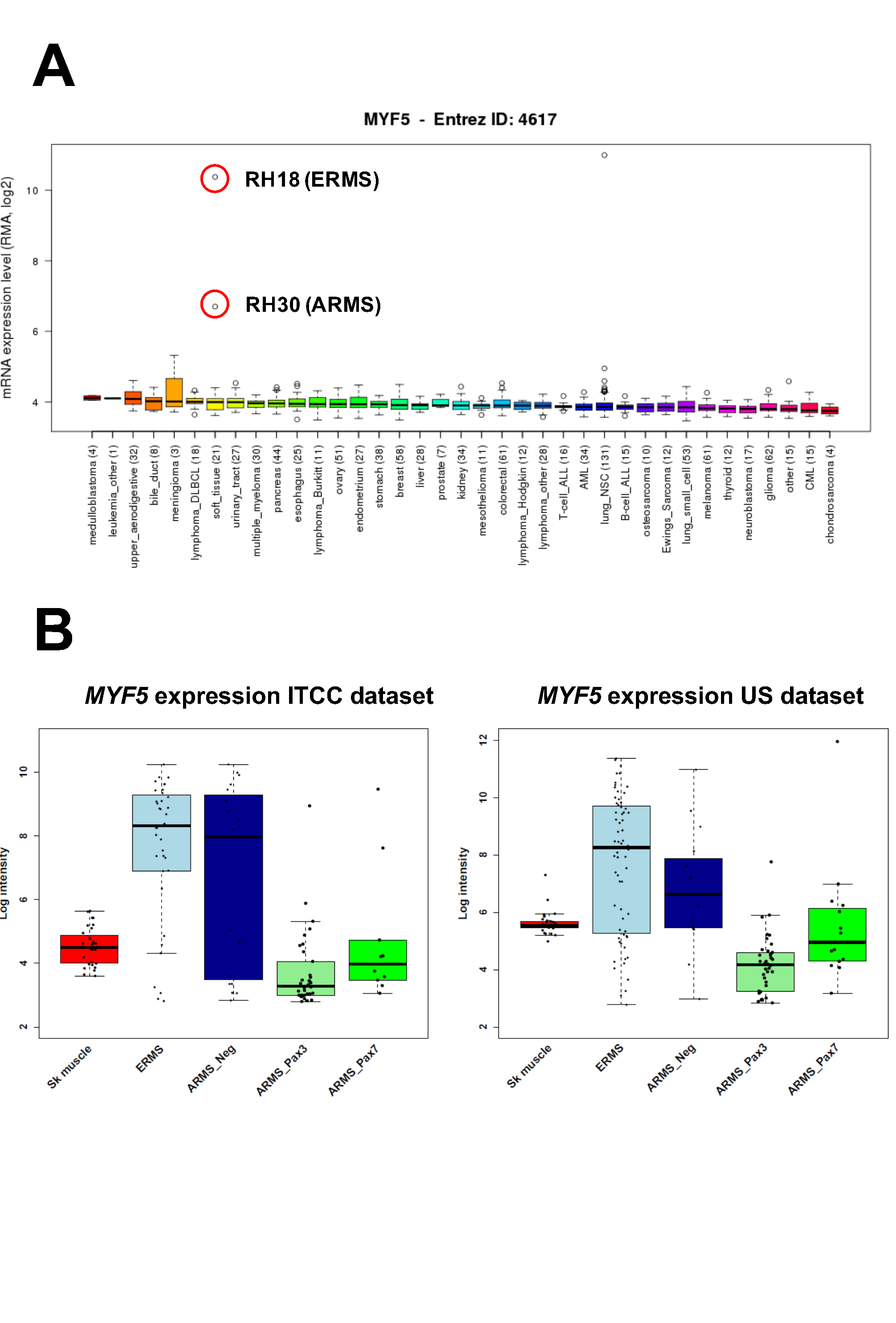

Supplement: Supplementary file 6 — Figure S4. Analyses of Myf5 expression in cancer cell lines and of MYF5 in different cohorts. (A) Myf5 expression in cancer cell lines; note the high‐level expression of Myf5 in the RH18 (ERMS) and RH30 (ARMS) cell lines. (B) MYF5 expression in human skeletal (sk.) muscle, ERMS, fusion gene‐negative ARMS (ARMS_Neg) and PAX3/7‐FOXO1‐positive ARMS (ARMS_Pax3, ARMS_Pax7) in the ITCC/CIT [33] and COG/IRSG cohorts [34] [file PATH-240-3-s007.tif]
