# Supplementary material for: The Hippo effector TAZ (WWTR1) transforms myoblasts and TAZ abundance is associated with reduced survival in embryonal rhabdomyosarcoma
Source: J Pathol. 2016 Aug 22;240(1):3–14. doi: 10.1002/path.4745 (PMC4995731; doi:10.1002/path.4745)
Supplement: Supplementary file 7 — Table S1. RT–qPCR primers used in this study Table S2. Name, description and ratio (firefly:Renilla) of the luciferase constructs used in dual‐luciferase reporter assays Table S3. Catalogue number and the target shRNA sequences of scramble control shRNA and TAZ shRNA [file PATH-240-3-s006.doc]

**Table S1.** RT–qPCR primers used in this study

| **Gene** | **Primers** |
| --- | --- |
| *WWTR1* (encoding human TAZ) | Forward 5′-ATTCGAATGCGCCAAGAG-3′  Reverse 5′-AACTGGGGCAAGAGTCTCAG-3′ |
| *Ctgf* | Forward 5′-TGACCTGGAGGAAAACATTAAGA-3′  Reverse 5′-AGCCCTGTATGTCTTCACACTG-3′ |
| *Myf5* | Forward 5′-CTGCTCTGAGCCCACCAG-3′  Reverse 5′-GACAGGGCTGTTACATTCAGG-3′ |
| *Phgdh* | Forward 5′-CAACCCCTGCTCGTATTCC-3′  Reverse 5′-TGGAGGTTTGGTAGGACAGC-3′ |
| *Psat1* | Forward 5′-CCGGTGGATGTTTCCAAGT-3′  Reverse 5′-GGTCATCCCGGACAATCA-3′ |
| *Psph* | Forward 5′-GCAAAGCTCAATATCCCAACA-3′  Reverse 5′-GGCTGCATCTCATCAAAACC-3′ |
| *YAP* | Forward 5′-TAGCCCTGCGTAGCCAGTTA-3′  Reverse 5′-TCATGCTTAGTCCACTGTCTGT-3′ |
| *CYR61* | Forward 5′-GGTCAAAGTTACCGGGCAGT-3′  Reverse 5′-GGAGGCATCGAATCCCAGC-3′ |
| *BIRC5* | Forward 5′-AGGACCACCGCATCTCTACAT-3′  Reverse 5′-AAGTCTGGCTCGTTCTCAGTG-3′ |
| *E2F1* | Forward 5′-CATCCCAGGAGGTCACTTCTG-3′  Reverse 5′-GACAACAGCGGTTCTTGCTC-3′ |
| *GAPDH* | Forward 5′-TGGAAGGACTCATGACCACA-3′  Reverse 5′-TTCAGCTCAGGGATGACCTT-3′ |

**Table S2.** Name, description and ratio (Firefly:*Renilla*) of the luciferase constructs used in dual-luciferase reporter assays

| **Luciferase construct** | | **Description** | **Ratio** | **Reference** |
| --- | --- | --- | --- | --- |
| 8XGTIIC | Luciferase reporter containing eight copies of TEAD binding sites (ACATTCCA) | | 25:1 | A gift from Stefano Piccolo (now deposited as Addgene plasmid no. 34615) |
| CTGF | Luciferase reporter of human CTGF promoter containing three TEAD binding sites (GGAATG) | | 10:1 | A gift from Kun-Liang Guan |
| Brachyury | Luciferase reporter containing two copies of T-box binding site (AGGTG) | | 10:1 | A gift from Malcolm Logan |

**Table** **S3.** Catalogue number and the target shRNA sequences of scramble control shRNA and TAZ shRNA

| **Catalogue/Clone No.** | **Target shRNA sequences** |
| --- | --- |
| SHC002 (Scramble shRNA) | CCGGCAACAAGATGAAGAGCACCAACTCGAGTTGGTGCTCTTCATCTTGTTGTTTTT |
| TRCN0000370007 (*TAZ* shRNA) | CCGGGCGTTCTTGTGACAGATTATACTCGAGTATAATCTGTCACAAGAACGCTTTTTG |

All plasmids were obtained from Sigma, UK.
